# Psychological predictors of insomnia, anxiety and depression in university students: potential prevention targets

**DOI:** 10.1192/bjo.2022.48

**Published:** 2022-04-19

**Authors:** Julia A. B. Lindsay, Niall M. McGowan, Nathan King, Daniel Rivera, Melanie Li, Jin Byun, Simone Cunningham, Kate E. A. Saunders, Anne Duffy

**Affiliations:** Department of Psychiatry, University of Oxford, UK; Department of Psychiatry, University of Oxford, UK; Department of Public Health Sciences, Queen's University, Canada; Department of Pharmacology and Toxicology, University of Toronto, Canada; and Department of Psychiatry, Queen's University, Canada; Department of Biology, Queen's University, Canada; Faculty of Health Sciences, Queen's University, Canada; Department of Biomedical and Molecular Sciences, Queen's University, Canada; Department of Psychiatry, University of Oxford, UK; Department of Psychiatry, University of Oxford, UK; and Department of Psychiatry, Queen's University, Canada

**Keywords:** Student mental health, insomnia, sleep disorders, anxiety disorders, depressive disorders

## Abstract

**Background:**

Perfectionism, low self-esteem and external locus of control are psychological constructs linked to insomnia, anxiety and depression. Examining how these constructs impact mental health and serve as risk factors for the development of clinically significant symptoms may help direct psychological support resources and preventative measures for university students.

**Aims:**

To longitudinally examine associations between the aforementioned psychological constructs and symptoms of insomnia, anxiety and depression in a large representative sample of first-year university students.

**Method:**

Electronic surveys including validated measures of the predictors and outcomes were emailed to all first-year undergraduate students at entry to a major Canadian university, and followed up on at conclusion of the academic year.

**Results:**

Compared with healthy sleepers, students screening positive for insomnia had lower self-esteem, higher self-evaluative perfectionism and increased external locus of control (all *P* < 0.001). Self-evaluative perfectionism (standardised *β* = 0.13, *P* < 0.01), self-esteem (*β* = −0.30, *P* < 0.001) and external locus of control (*β* = 0.07, *P* = 0.02) measured at entry were significantly associated with insomnia symptoms at follow-up. Insomnia symptoms at entry were strong predictors of symptoms of depression (*β* = 0.15, *P* < 0.001) and anxiety (*β* = 0.16, *P* < 0.001) at follow-up, even after controlling for baseline symptoms of those disorders.

**Conclusions:**

Perfectionism, low self-esteem and external locus of control may predispose the development of insomnia symptoms in university students. In turn, insomnia symptoms appear to be robust predictors for depressive and anxiety symptoms. Sleep may be an important prevention target in university students.

During the transition to university, stressors such as increased academic workload, added responsibilities and autonomy, and living with peers contribute to heightened risk of irregular sleep–wake patterns and poor sleep quality in students.^1^ When these symptoms co-occur with daytime fatigue, long sleep-onset latency, and difficulty maintaining sleep, they constitute symptoms of insomnia disorder.^2^ Insomnia disorder is common in university students, with estimates ranging from 10 to 14% of students meeting diagnostic criteria.^1,3^ Insomnia is associated with a host of negative outcomes, including physical illness, reduced well-being and worsened academic performance.^4,5^ Increasingly, evidence suggests that untreated insomnia symptoms contribute to the development of clinically significant depression and anxiety, both of which are associated with reduced academic performance and are the most common mental health problems reported by university students.^5–7^ The existing literature addressing insomnia in students is largely cross-sectional, and thus does not disentangle causal from associated factors, nor capture longitudinal changes in symptoms as students progress through their university studies.

## Risk factors for insomnia

**Fig. 1:**
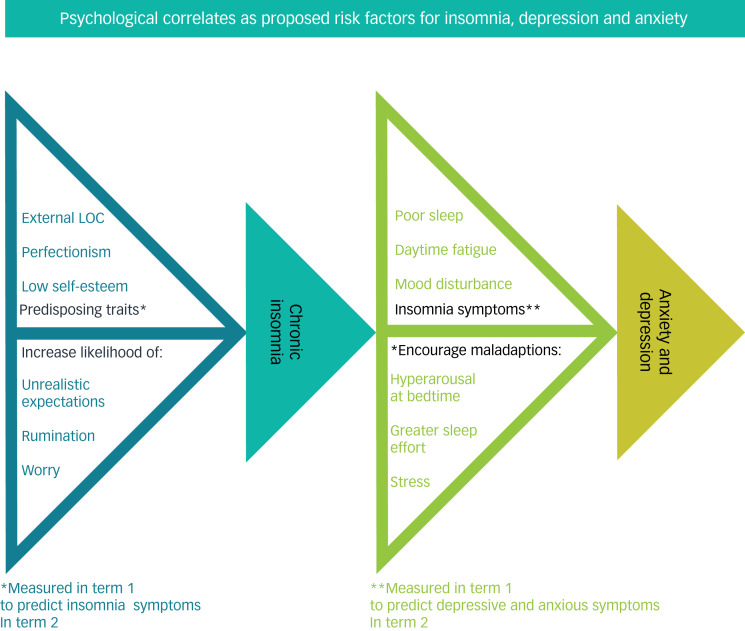
Proposed model of the effects of external locus of control (LOC), perfectionism and low self-esteem on the development of insomnia, anxiety and depression. Based on existing models of insomnia.^13,21^

Prevailing models of insomnia suggest that the disorder arises in response to predisposing, precipitating and perpetuating factors. Insomnia is believed to be precipitated by an acute event such as a period of stress, and may be perpetuated by chronic factors like poor sleep habits, a bedroom environment that is non-conducive to uninterrupted sleep or dysfunctional beliefs and expectations about sleep.^8^ The tendency for worry or rumination in response to stress is a predisposing factor for insomnia, which may lead to greater arousal before sleep or encourage greater sleep effort.^8,9^ Worry and rumination are correlated with perfectionism and low self-esteem.^10^ The former is defined as the maladaptive habit of judging oneself against unreasonably high self-imposed standards, which eventually cannot be met and result in self-criticism.^11^ Perfectionism has been linked to poor sleep and increased symptoms of depression and anxiety.^12,13^ Self-esteem, defined as positive appraisal of oneself, is lower in poor sleepers even after controlling for sleep duration and depressive symptoms, and is also associated with anxiety and depression.^14–16^ External locus of control refers to the tendency to attribute one's life events to luck, fate or external influences rather than oneself. External locus of control has been associated with anxiety and depression, and may be linked to insomnia and low academic performance.^17–20^ In this analysis, we examine perfectionism, self-esteem and locus of control as predictors of symptoms of insomnia, depression and anxiety. The proposed links between these concepts are shown in Figure 1. All three psychological constructs were chosen not only because they are associated with these common mental health outcomes, but also because they are amenable to cognitive behavioural therapy interventions.^19,22,23^

## Aims

We sought to explore the direct links between the psychological constructs of perfectionism, self-esteem and locus of control, and insomnia, and the association between insomnia and symptoms of anxiety and depression, in undergraduates over their first year of study at a major Canadian university. We hypothesised that perfectionism, low self-esteem and external locus of control contribute to the development of insomnia symptoms. A secondary hypothesis was that insomnia symptoms in conjunction with the aforementioned psychological constructs would predict severity of depressive and anxiety symptoms.

## Method

### Procedure

The authors assert that all procedures contributing to this work comply with the ethical standards of the relevant national and institutional committees on human experimentation and with the Helsinki Declaration of 1975, as revised in 2008. All procedures involving human participants were approved by the Queen's University Health Sciences and Affiliated Teaching Hospitals Research Ethics Board (approval number PSIY-608-18). Informed consent was obtained from all participants before survey completion.

The U-Flourish Student Well-Being study is a longitudinal repeated-measures study of students at Queen's University. Information about sampling and recruitment for the study has been described elsewhere.^5,24,25^ Briefly, student-led campus engagement campaigns raised awareness and encouraged participation among first-year students at the start of each academic year the survey was conducted. All eligible first-year students at the university were emailed a link to complete the entry survey in their third week of the first term, which remained available for 2–3 weeks. An end-of-year survey was sent out 3 weeks before the start of the final examination period at the end of the academic year, and was also open for a 3-week period. As incentive to participate, students were entered into a draw for one of five iPads for each survey they responded to. Additionally, all students who completed the autumn survey were emailed a voucher for a free drink at a café on campus.

### Participants

**Fig. 2:**
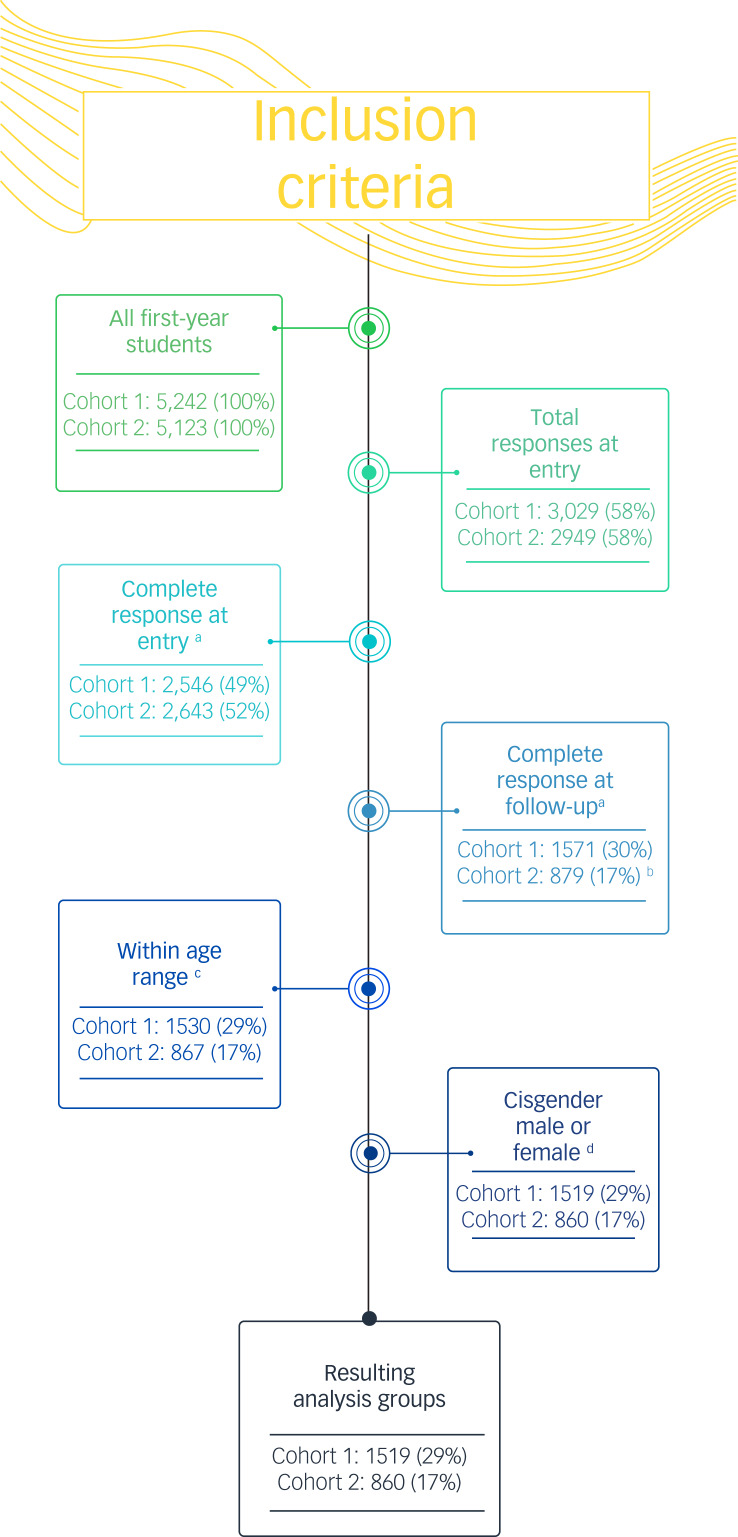
Number of responses included for all inclusion criteria. Percentages are of all first-year students. ^a^Variables of interest were complete. ^b^Reduced response rate likely because of COVID-19. ^c^Aged 17-22 years, inclusive. ^d^Excluding self-reported transgender, genderfluid, non-binary or other gender identity.

Data were obtained from two independent cohorts of first-year students entering Queen's University in the academic years 2018–2019 (*n* = 1519) and 2019–2020 (*n* = 860), hereafter labelled cohorts 1 and 2, respectively. Analyses were restricted to participants aged 17–22 years, as this range constituted 99% of the participants and best represented typical first-year university students. Compared with the group of all eligible students, the analysis group was significantly younger (17.9 *v.* 18.5, *P* < 0.01) and significantly more likely to be female (74% *v.* 58%, *χ*^2^-test *P* < 0.01). Distribution by academic programme was similar between groups. There was insufficient statistical power to include transgender, genderfluid and other gender diverse participants (<1%) in these statistical analyses. Figure 2 shows the number of responses included for each inclusion criteria.

### Measures

Supplementary Table 1, available at https://doi.org/10.1192/bjo.2022.48, outlines the timing of events and measures.

#### Measured at entry to first year

##### Self-reported diagnosis history

Participants selected relevant responses to the following question: ‘Have you ever been diagnosed with any of the following mental health conditions or learning problems?’ In this analysis, reports of ‘mood disorder (e.g. depression, dysthymia, bipolar disorder)’, ‘anxiety disorder (e.g. post-traumatic stress disorder, obsessive–compulsive disorder, panic disorder, simple phobia, social phobia, generalised anxiety disorder, agoraphobia)’ or ‘sleep disorder (e.g. insomnia)’ were used as control variables.

##### Clinical Perfectionism Questionnaire

The Clinical Perfectionism Questionnaire (CPQ) is a 12-item scale self-report measure of a clinically driven definition of perfectionism.^11^ Scores range from 12 to 48, with higher scores indicating higher clinical perfectionism. The CPQ was split into two factors as proposed by Dickie et al: personal standards (range: 6–24), which correspond to the setting, checking and upward trending of self-imposed ideals; and self-evaluative concerns (range: 4–16), which captures distress as a response to perceived failure.^27^ Cronbach's *α* was calculated as 0.73 for personal standards, and 0.59 for evaluative concerns.

##### German Socio-Economic Panel short scale for locus of control

The German Socio-Economic Panel scale contains two subscales: internal locus of control (three items, range: 0–18) and external locus of control (five items, range: 0–30).^26^ The former measures perceived personal control over the events of the respondent's life; the latter measures perceived impact of external forces, including fate, luck, other people and inborn ability. The internal subscale had a Cronbach's *α* of 0.48, and the external subscale had a Cronbach's *α* of 0.71. In the original scale, higher scores in each subscale indicate lower attribution either to personal will (internal locus of control) or external forces (external locus of control). However, in this analysis the measures were reverse-coded such that higher scores indicated stronger attribution.

##### Rosenberg Self-Esteem Scale

The Rosenberg Self-Esteem Scale is a ten-item self-report measure of self-esteem that was originally designed for use with high school students but has since been validated in a variety of populations.^28^ Scores range from 10 to 40, with higher scores indicating higher self-esteem.

#### Measured at entry and completion of the first year

##### Sleep Condition Indicator

The Sleep Condition Indicator (SCI) is an eight-item screening tool for insomnia symptoms based on the DSM-5 criteria, with possible scores ranging from 0 to 32 and a score of 16 representing the threshold for clinically significant symptoms.^9^ For this analysis, continuous scores were reverse-coded such that higher scores represent more severe insomnia symptoms. Questions address factors such as sleep continuity, severity of insomnia and daytime impairment symptoms. The SCI has strong internal consistency (Cronbach's *α* = 0.86) and convergence with other insomnia screening tools.^9^ In this analysis, the baseline SCI measurement was used as a continuous predictor of depression and anxiety symptoms measured at the end of the year, whereas the end of year SCI score was used as a continuous outcome predicted by the psychological constructs measured at baseline.

##### Patient Health Questionnaire-9

The Patient Health Questionnaire-9 (PHQ-9) is a commonly used screening tool that measures depressive symptoms by using nine items, each scored on a scale of 0 (‘not at all’) to 3 (‘nearly every day’). For the primary analysis, the PHQ-9 scores were modelled as continuous (range: 0 to 27), although a clinically significant cut-off score of ≥10 was used in the Supplementary materials. The PHQ-9 has demonstrated strong reliability and validity in a variety of samples.^29,30^

##### Generalised Anxiety Disorder-7 assessment

The Generalised Anxiety Disorder-7 (GAD-7) assessment is a screening tool used to measure generalised anxiety symptoms on a 0–21 scale. For the primary analysis, GAD-7 scores were modelled as continuous, although a clinically significant cut-off score of ≥10 was used in the Supplementary materials. It has been shown to have strong validity and reliability.^31,32^

### Statistical analysis

Person-mean imputation was used for scale data when a single item was missing. If more than one item was missing from a scale, the entire scale was coded as missing. As none of the variables of interest had >20% of the scale results missing, a complete-case analysis was used. Complete-case analysis was chosen because data tended to be either complete or missing for all scales (rather than a single scale), because of students not responding to the end-of-year questionnaire. In this situation, complete-case analysis is preferred.^33^ PHQ-9 and GAD-7 scores were both square-root adjusted to compensate for positive skew.

For the purposes of descriptive analysis, proportions of students meeting the threshold of clinically significant scores based on established cut-offs on the SCI, PHQ-9 and GAD-7 were compared at entry and conclusion of the first year of university. Comparisons between these proportions were made with McNemar's *χ*^2^-tests.^34^ Comparisons between the groups screening positive and negative for the above disorders were made with Welch's independent sample *t*-tests.^35^ Correlations between psychological constructs were investigated with Spearman's rho.^36^

Associations between psychological constructs measured at entry to university and end-of-year SCI scores were examined with hierarchical regressions, meaning covariates were entered in a separate step ahead of predictors. This method allowed us to control for the effects of variables such as gender, pre-existing diagnoses and baseline symptoms.^37^ Hierarchical regression is also appropriate for correlated variables, which was important as symptoms of insomnia, depression and anxiety were correlated.^37^ Further hierarchical regressions used psychological construct measurements and SCI scores at entry to predict continuous PHQ-9 and GAD-7 scores at the end of first year. Linear regression was chosen instead of logistic as we felt that continuous measures of the psychological constructs would better predict continuous symptom measures than binary screening results. This approach is also less pathologizing, an important consideration in student mental health. However, in further analysis, logistic regression was used to examine the associations between predictors at entry and positive disorder screens at completion of first year; these models are reported in the Supplementary materials.

Separate models were run for cohort 1 (2018–2019) and cohort 2 (2019–2020). The data were not combined because of changes in survey content between years: locus of control was only measured in cohort 1 and perfectionism was only measured in cohort 2. This change was a result of student feedback suggesting that perfectionism was an important measure; to keep survey length consistent from year to year, the locus of control measure was replaced by the CPQ. Models examining SCI scores as the outcome were adjusted for gender and existing sleep disorder diagnoses; those with PHQ-9 scores as the outcome were adjusted for gender and lifetime mood disorder diagnoses. Finally, models with GAD-7 scores as the outcome were adjusted for gender and lifetime anxiety disorder diagnoses. The variance inflation factor was <2.5 for all covariates in all models, suggesting acceptable collinearity.

All statistical analyses were conducted with R for MacOS version 4.0.3 (R Core Team (2020), Vienna, Austria; see https://www.R-project.org/). Results were reported as statistically significant where a *P*-value of <0.05 was detected. Linear regressions used lm() and logistic regressions used glm().

## Results

### Descriptive analysis

In cohort 1 (2018–2019), 3069 first-year students responded, representing 58% of the eligible first-year students across programmes of study. In cohort 2 (2019–2020), 2995 first-year students responded, representing 62% of the first-year undergraduate population. Survey participants had a mean age of 18.2 years in cohort 1 (s.d. 1.9) and 18.1 years in cohort 2 (s.d. 1.2). They were also more likely to identify as female than the group of all eligible students (65 *v.* 58%, *t*(6063) = 19.0, *P* < 0.001). Study participants were most likely (34%) to be enrolled in an arts, humanities or social science programme. Life and physical sciences students made up 28% of the sample and a further 16% were studying engineering and applied science.

In this complete-case analysis, 1519 and 860 students from cohorts 1 and 2, respectively, had complete data at both time points on the variables of interest (shown in Fig. 2). As listed in Table 1, the mean outcome scores for insomnia, depression and anxiety symptoms at completion of the year (*P* = 0.89, *P* = 0.95 and *P* = 0.77, respectively) were not significantly different between cohorts 1 and 2.

**Table 1:** Prevalence of reported lifetime mental health disorders and clinically significant symptoms at entry to university and completion of first year, by cohort

Outcome measure	Score at entry to first year, mean (s.d.)	Score at end of first year, mean (s.d.)	Proportion at entry to university, *n* (%)	Proportion at end of first year, *n* (%)	Difference in proportions, entry versus year end (*P-*value)
Cohort 1 analysis group (2018–2019)			1519 (100.0)		
Female	–	–	1108 (72.9)	–	–
Lifetime sleep disorder^a^	–	–	43 (2.8)	67 (4.4)	<0.001
Lifetime mood disorder^a^	–	–	149 (9.8)	183 (12.0)	<0.001
Lifetime anxiety disorder^a^	–	–	258 (17.0)	307 (20.2)	<0.001
Clinically significant insomnia symptoms^b^	9.7 (6.5)	11.8 (7.1)	304 (20.0)	447 (29.4)	<0.001
Clinically significant depression symptoms^c^	6.9 (5.7)	8.6 (6.5)	410 (27.0)	557 (36.7)	<0.001
Clinically significant anxiety symptoms^d^	7.5 (5.6)	8.5 (6.1)	487 (32.1)	590 (38.8)	<0.001
Cohort 2 analysis group (2019–2020)			860 (100.0)		
Female	–	–	640 (74.4)	–	–
Lifetime sleep disorder^a^	–	–	28 (3.3)	44 (5.1)	<0.001
Lifetime mood disorder^a^	–	–	103 (12.0)	119 (13.8)	<0.001
Lifetime anxiety disorder^a^	–	–	186 (21.6)	204 (23.7)	<0.001
Clinically significant insomnia symptoms^b^	11.4 (7.1)	11.8 (7.5)	239 (27.8)	284 (33.0)	<0.001
Clinically significant depression symptoms^c^	7.8 (6.1)	8.6 (6.3)	287 (33.3)	318 (37.0)	0.03
Clinically significant anxiety symptoms^d^	7.6 (5.6)	8.4 (5.6)	274 (31.9)	328 (38.3)	<0.001

*P*-values are from McNemar's *χ*^2^-test.

a. Entry to university: lifetime student-reported diagnoses up until entry to university. Completion of first year: lifetime student-reported diagnoses including new diagnoses over the first year of university.

b. Students screening positive on the reverse-coded Sleep Condition Indicator, using a threshold score of ≥16.

c. Students screening positive on the Patient Health Questionnaire-9, using a threshold score of ≥10, before square-root transformation.

d. Students screening positive on the Generalised Anxiety Disorder-7, using a threshold score of ≥10, before square-root transformation.

In each cohort, significantly more students screened positive for insomnia, depression and anxiety disorders at the completion compared with beginning of the academic year (Table 1). There were also significant increases in the number of student-reported sleep, mood and anxiety disorder diagnoses by the conclusion of the academic year.

#### Differences between insomnia and healthy sleep groups

There were significant differences in baseline psychological construct scores between the student groups who screened positive for insomnia (above the SCI threshold score of 16) compared with those who did not (Table 2). Overall, students who screened positive for insomnia tended to have more external locus of control, lower self-esteem and higher evaluative concerns as a dimension of perfectionism. Scores on the personal standards subscale of perfectionism were not significantly different.

**Table 2:** Comparisons of psychological construct scores between groups screening positive and negative for insomnia at baseline (entry to university)

	Healthy sleep group,^a^ mean (s.d.)	Insomnia group,^b^ mean (s.d.)	*t*-Test statistic^c^	95% CI for difference between means^c^	*P*-value^c^
Self-esteem,^d^ cohorts 1 and 2	19.94 (5.27)	15.61 (5.47)	−25.2	−4.67 to −3.99	<0.001
External locus of control,^e^ cohort 1	13.79 (5.11)	16.15 (5.13)	9.76	1.89–2.83	<0.001
Evaluative concerns,^f^ cohort 2	9.98 (2.32)	11.61 (2.28)	16.53	1.44–1.83	<0.001
Personal standards,^f^ cohort 2	16.75 (3.09)	16.90 (3.12)	1.11	−0.11 to 0.41	0.27

a. Negative screen for insomnia, using the Sleep Condition Indicator with threshold score of 16.

b. Positive screen for insomnia, using the Sleep Condition Indicator with threshold score of 16.

c. Value from Welch's *t*-test.

d. Rosenberg Self-Esteem Scale.

e. German Socio-Economic Panel external locus of control scale.

f. Subscale of Clinical Perfectionism Questionnaire.

#### Associations between psychological constructs

Rosenberg Self-Esteem Scale scores were negatively correlated with external locus of control (Spearman's rho = −0.48, *P* < 0.001). Self-esteem was also strongly negatively correlated with the evaluative concerns subscale scores (Spearman's rho = −0.63, *P* < 0.001), but showed no statistically significant correlation with the personal standards subscale scores.

### Predictive hierarchical regression model

In Table 3, adjusted *R*^2^ values indicate that 14% (cohort 1) and 20% (cohort 2) of the variation in insomnia symptoms at completion of first year was explained by the combination of reported lifetime sleep disorder diagnoses and psychological constructs at entry to university. Internal and external locus of control scores had opposite associations with insomnia symptoms: external locus of control was associated with more insomnia symptoms, whereas internal locus of control, although not significant, was associated with fewer symptoms. The evaluative concerns dimension of perfectionism was a significant predictor of increased insomnia symptoms, whereas personal standards was not. Self-esteem was a stronger predictor of reduced insomnia symptoms than any other psychological or clinical risk factor tested, including a self-reported lifetime (prior) sleep disorder.

**Table 3:** Multivariable linear regression predicting associations between psychological risk factors measured at entry to university and insomnia symptoms measured at completion of first year^a^

Sleep Condition Indicator	Cohort 1 (2018–2019)					Cohort 2 (2019–2020)				
	Δ*R*^2^	*β*	s.e. *β*	Standardised *β*	*P-*value	Δ*R*^2^	*β*	s.e. *β*	Standardised *β*	*P-*value
Control variables	0.04					0.05				
Constant		23.27	2.31		<0.001		23.83	3.01		<0.001
Female		1.70	0.41	0.10*	<0.001		2.07	0.57	0.12*	<0.001
Lifetime sleep disorder^b^		7.32	1.08	0.17*	<0.001		7.94	1.40	0.19*	<0.001
Adjusted model	0.10					0.14				
Constant		28.34	2.52		<0.001		25.01	3.57		<0.001
Female		0.73	0.39	0.05	0.06		0.25	0.55	0.01	0.64
Lifetime sleep disorder^b^		5.42	1.03	0.13*	<0.001		5.99	1.30	0.14*	<0.001
Locus of control										
Internal^c^^,^^d^		−0.09	0.08	−0.03	0.26		–	–	–	–
External^c^^,^^d^		0.10	0.04	0.07*	0.02		–	–	–	–
Perfectionism										
Personal standards^e^^,^^f^		–	–	–	–		0.05	0.08	0.02	0.55
Evaluative concerns^e^^,^^f^		–	–	–	–		0.40	0.13	0.13*	< 0.01
Self-esteem^g^		−0.39	0.04	−0.29*	<0.001		−0.38	0.05	−0.30*	<0.001
Adjusted *R*^2^ for final model	0.14					0.20				

a. Continuous value measured by the Sleep Condition Indicator, with high scores representing worse insomnia symptoms.

b. Student-reported at entry to university.

c. No locus of control measures available for cohort 2.

d. Subscale of German Socio-Economic Panel locus of control scale.

e. No perfectionism measures available for cohort 1.

f. Subscale of Clinical Perfectionism Questionnaire.

g. Rosenberg Self-Esteem Scale.

* *P* < 0.05.

Table 4 shows that 35% (cohort 1) and 50% (cohort 2) of the variation in symptoms of depression at completion of first year is explained by models that include gender, lifetime mood disorder, insomnia symptoms and the examined psychological risk factors measured at entry to university. Internal and external locus of control had opposite standardised associations with depression symptom: just as in the previous model, external locus of control was associated with more symptoms of depression and internal locus of control was associated with fewer symptoms of depression. As in the insomnia symptom prediction model, evaluative concerns significantly predicted worsened depression symptoms, whereas high personal standards did not. Aside from depression symptoms at entry, insomnia symptoms at entry were the strongest predictor of end-of-year depression symptoms.

**Table 4:** Multivariable linear regression predicting associations between psychological risk factors measured at entry to university and symptoms of depression measured at completion of first year^a^

PHQ-9	Cohort 1^b^ (2018–2019)					Cohort 2^c^ (2019–2020)				
	Δ*R*^2^	*β*	s.e. *β*	Standardised *β*	*P-*value	Δ*R*^2^	*β*	s.e. *β*	Standardised *β*	*P*-value
Control variables	0.31					0.47				
Constant		1.10	0.11		<0.001		0.52	0.15		<0.001
Female		0.12	0.06	0.04	0.05		0.09	0.07	0.03*	0.23
Lifetime mood disorder^d^		0.39	0.09	0.09*	<0.001		0.41	0.10	0.11*	<0.001
Baseline depression^e^		0.55	0.02	0.52*	<0.001		0.62	0.03	0.63*	<0.001
Adjusted model	0.03					0.03				
Constant		1.81	0.26		<0.001		1.22	0.36		<0.001
Female		0.07	0.07	0.02	0.25		0.05	0.07	0.02*	0.49
Lifetime mood disorder^d^		0.24	0.10	0.06*	0.01		0.35	0.10	0.09*	<0.001
Baseline depression^e^		0.36	0.03	0.34*	<0.001		0.43	0.04	0.44*	<0.001
Locus of control										
Internal^f^		0.00	0.01	−0.01	0.80	–	–	–	–	–
External^f^		0.02	0.01	0.06*	<0.01	–	–	–	–	–
Perfectionism										
Personal standards^g^		–	–	–	–		0.01	0.01	0.03	0.23
Evaluative concerns^g^		–	–	–	–		0.05	0.02	0.10*	<0.01
Self-esteem^h^		−0.03	0.01	−0.14*	<0.001		−0.02	0.01	−0.11*	<0.01
Baseline insomnia^i^		0.03	<0.01	0.15*	<0.001		0.02	0.01	0.12*	<0.001
Adjusted *R*^2^ for final model	0.35					0.50				

PHQ-9, Patient Health Questionnaire-9.

a. Continuous value measured by PHQ-9 at follow-up, square-root adjusted.

b. *P*<0.05.

c. Student-reported at entry to university.

d. Measured by the PHQ-9 at entry.

e. Measured by the German Socio-Economic Panel locus of control subscale at entry. No data available for Cohort 2.

f. Measured by the Clinical Perfectionism Questionnaire subscale at entry. No data available for Cohort 1.

g. Measured by the Rosenberg Self-Esteem Scale at entry.

h. Measured by the Sleep Condition Indicator, reverse-coded at entry.

The PHQ-9 contains one item that addresses sleep difficulties: ‘Trouble falling/staying asleep or sleeping too much’. To determine whether this item was affecting the regressions in Table 4, the same analyses were repeated with the sleep item removed from the PHQ-9 scores. The resulting adjusted *R*^2^ values were nearly identical to those from the regression with the sleep item included: 0.35 (cohort 1, unchanged) and 0.49 (cohort 2, 0.01 decrease). The full regressions are included in Supplementary Tables 2 and 3.

Table 5 shows that 37% (cohort 1) and 46% (cohort 2) of the variation in end-of-year anxiety symptoms can be attributed to gender, pre-existing anxiety disorder diagnoses and symptoms, pre-existing insomnia symptoms and psychological constructs. As with models predicting insomnia and depression symptoms, internal and external locus of control had opposite associations with anxiety, with external locus of control significantly associated with increased anxious symptoms. This was the only model in which personal standards significantly predicted worsened symptom severity. Aside from anxiety symptoms at entry, insomnia symptoms at entry were the strongest predictor of end-of-year anxiety symptoms.

**Table 5:** Multivariable linear regression predicting associations between psychological risk factors measured at entry to university and symptoms of anxiety measured at completion of first year^a^

GAD-7	Cohort 1^b^ (2018–2019)					Cohort 2^c^ (2019–2020)				
	Δ*R*^2^	*β*	s.e. *β*	Standardised *β*	*P-*value	Δ*R*^2^	*β*	s.e. *β*	Standardised *β*	*P-*value
Control variables	0.32					0.42				
Constant		0.86	0.11		<0.001		0.90	0.12		<0.001
Female		0.24	0.06	0.09*	<0.001		0.22	0.07	0.09*	<0.01
Lifetime anxiety disorder^d^		0.37	0.07	0.11*	<0.001		0.39	0.08	0.14*	<0.001
Baseline anxiety^e^		0.52	0.02	0.49	<0.001		0.53	0.03	0.55*	<0.001
Adjusted model	0.04					0.04				
Constant		1.40	0.25		<0.001		0.27	0.30		0.37
Female		0.21	0.06	0.08*	<0.001		0.18	0.07	0.07*	0.01
Lifetime anxiety disorder^d^		0.25	0.07	0.08*	<0.001		0.30	0.07	0.11*	<0.001
Baseline anxiety^e^		0.35	0.03	0.33	<0.001		0.37	0.03	0.38*	<0.001
Locus of control										
Internal^f^		−0.01	0.01	−0.01	0.63		–	–	–	–
External^f^		0.02	0.01	0.08*	<0.01		–	–	–	–
Perfectionism										
Personal standards^g^		–	–	–	–		0.04	0.01	0.10*	<0.001
Evaluative concerns^g^		–	–	–	–		0.04	0.02	0.09*	0.01
Self-esteem^h^		−0.03	0.01	−0.11*	<0.001		−0.01	0.01	−0.07	0.06
Baseline insomnia^i^		0.03	<0.01	0.16*	<0.001		0.02	<0.01	0.14*	<0.001
Adjusted *R*^2^ for final model	0.37					0.46				

GAD-7, Generalised Anxiety Disorder-7.

a. Continuous value measured by the GAD-7 at follow-up, then square-root adjusted.

b. *P* < 0.05.

c. Student-reported at entry to university.

d. Measured by the GAD-7 at entry.

e. Measured by the German Socio-Economic Panel locus of control subscale at entry. No data available for Cohort 2.

f. Measured by the Clinical Perfectionism Questionnaire subscale at entry. No data available for Cohort 1.

g. Measured by the Rosenberg Self-Esteem Scale at entry.

h. Measured by the Sleep Condition Indicator, reverse-coded at entry.

i. *P* < 0.05.

## Discussion

### Prevalence of insomnia, depression and anxiety

At the end of first year, 29–33% of participants screened positive for insomnia, 36–37% for depression and 38–39% for anxiety. Clinical levels of insomnia appeared to be considerably more prevalent in this sample than the 10–14% reported by the extant literature on university students.^1,3^ Depression was also more prevalent than the 14–31% suggested in reviews.^38,39^ Finally, anxiety prevalence is not a well-established value in university students, with estimates ranging from 4–28%, but this sample also exceeds these.^39,40^ There are several likely contributors to elevated prevalence rates in this sample. First, we used clinical symptom threshold scores instead of asking students to report formal diagnoses. Also, this sample is mostly female and all three disorders are more common in women.^41,42^ Additionally, increased awareness and de-stigmatisation of mental health in recent years may drive increased symptom reporting compared with the past decade.^43^

### Psychological predictors of insomnia

Reduced self-esteem, higher evaluative concerns as a dimension of perfectionism and external locus of control measured at entry to university were significant predictors of insomnia, anxiety and depressive symptom severity at completion of first year in undergraduate students. The other factor of perfectionism, which measures tendency to set unreasonably high personal standards, was also a significant predictor of anxiety symptoms but not insomnia or depression. Finally, insomnia symptoms at entry to university were significant predictors of symptoms of depression and anxiety at the end of year.

### Mechanisms of predisposing factors

Candidate psychological risk factors studied here were each significant predictors of symptoms of insomnia, depression and anxiety. Self-esteem emerged as a stronger predictor of symptoms than the other psychological constructs, with higher self-esteem predicting fewer symptoms of insomnia, anxiety and depression. This aligns with previous evidence that self-esteem is associated with depression.^16^ To our knowledge, self-esteem has not been proposed as a predisposing factor for insomnia, but evidence from this survey study of university students supports the possibility. A speculative pathway may be increased worry and rumination because of the associated psychological distress of low self-esteem, whereas high self-esteem has protective effects.^44^ Further work is required to determine whether low self-esteem plays a causal role in the development of insomnia and anxiety.

Responding to calls for further investigation into the link between perfectionism and insomnia, we found that only one proposed factor of clinical perfectionism (evaluative concerns) predicted insomnia and depression symptoms, whereas anxiety symptoms were predicted by both evaluative concerns and high personal standards.^10^ This supports the hypothesis that the CPQ contains two distinct factors, as advanced by Dunkley et al and Dickie et al.^13,27^ It also aligns with the theory that self-evaluative concerns distinguish between maladaptive and normative perfectionism.^13^ Self-evaluative concerns can manifest as feelings of regret or self-directed scrutiny, which may predispose insomnia via increased cognitive arousal at bedtime.^21^ It is not clear whether high personal standards predispose anxiety independently or only in conjunction with evaluative concerns; indeed, some literature suggests high personal standards are characteristic of adaptive perfectionism.^45^ Finally, findings regarding external locus of control aligned with existing links to depression and anxiety and add to sparse literature on the link with insomnia. To our knowledge, only one other study has found a direct association between insomnia and locus of control.^18^ External locus of control may contribute to the development of insomnia because of feelings of learned helplessness and reduced self-determination, which are also the hypothesised pathways for the relationship between locus of control and depression.^18,46^

### Insomnia and the development of depression and anxiety

Insomnia symptoms at entry to university were one of the strongest predictors of anxious and depressive symptoms at the end of the year, even after baseline depression and anxiety was controlled for. This finding supports the hypothesis that insomnia is a risk factor for the development of these common mental health problems.^6,7^ This is notable given the prevalence of insomnia in university students: in both cohorts, about 30% of students reported clinically significant insomnia symptoms by the conclusion of their first year. Promisingly, cognitive–behavioural therapy for insomnia, has been shown to reduce both dysfunctional beliefs about sleep and depressive symptoms and thus may be worthy of future study as an early intervention for students.^47,48^ One of the mechanisms of cognitive–behavioural therapy for insomnia is reduction of repetitive negative thinking and pre-sleep arousal, which are our hypothesised behavioural links between insomnia and the psychological constructs of self-esteem, self-evaluative concerns and locus of control (Fig. 1).^49^

### Implications

These findings support widespread dissemination of evidence-based sleep guidance among first-year university students, who tend to have significantly greater ‘social jetlag’ than returning students.^4^ Social jetlag describes the discrepancy between sleep timing on work days and rest days, which is associated with worsened well-being and increased stimulant use.^50^ Descriptive analysis of this sample demonstrated that sleep disorders may not be as readily recognised as other common disorders, given that lifetime sleep diagnoses were reported less commonly than anxiety and depression, yet nearly a third of students screened positive for insomnia symptoms. These symptoms may continue after university: insomnia is the most common mental health problem in adults, and is persistent if left untreated.^9,46,51^ Eighty per cent of Canadian undergraduates report not seeking help for sleep problems, but fortunately effective sleep education programmes have already been developed.^52,53^ Psychological risk factors such as self-esteem, locus of control and perfectionism may also be identifiable and important prevention targets in help-seeking students with symptoms of insomnia, anxiety and depression.^19,22,23^

### Strengths and limitations

Our findings are notable in that they are longitudinal over 2 years, with consistent results in the repeated measures, especially the association between self-esteem and insomnia symptoms. Unlike many other studies of insomnia in student populations, this work uses a validated questionnaire that goes beyond sleep problems, to capture all DSM-5 criteria for insomnia, including daytime impairment.^6,9^ Furthermore, the locus of control and perfectionism measures were separated into subscales, which allowed disentangling of the associations between individual factors. However, the results are reliant on self-report measures, which only have moderate accuracy, particularly for self-reported insomnia.^54^ The analysis group was limited in scope as it was self-selecting, younger, much more likely to be female and only included cisgender students; further work is required to confirm the effects of these psychological constructs on the mental health of transgender, genderfluid and non-binary students. Attrition, particularly in cohort 2, is another limitation. This is likely attributable to the effects of the COVID-19 pandemic, as the university cancelled in-person class on 13 March 2020 and the follow-up survey was open from 16 to 30 March 2020. Students were likely preoccupied, and no in-person engagement events were possible. Despite the reduced response rate, we suspect that the effects of remote learning were not captured in this data-set because of the compressed timeline. Furthermore, there were no significant differences in proportions of end-of-year positive disorder screens between 2019 and 2020, and the results of this analysis were consistent between cohorts.

In conclusion, low self-esteem, self-evaluative perfectionism and external locus of control predicted insomnia, depressive and anxious symptoms. Insomnia symptoms at entry to university were robust predictors of future depressive and anxiety symptoms. Students with positive insomnia screens had significantly lower self-esteem, more self-evaluative perfectionist concerns and more external locus of control, compared with students with healthy sleep. These results may help identify students that could benefit from early intervention treatment for sleep problems, which may in turn reduce the risk of developing depression or anxiety during their undergraduate studies. Early identification of insomnia, low self-esteem, self-critical perfectionism and external locus of control should be prioritised, as these appear to be important prevention targets in symptomatic help-seeking students.

## Data Availability

The data that support the findings of this study are available from the corresponding author, A.D., upon reasonable request.

## Supplementary material

For supplementary material accompanying this paper visit http://doi.org/10.1192/bjo.2022.48. click here to view supplementary material
