# Supplementary material for: Psychological predictors of insomnia, anxiety and depression in university students: potential prevention targets
Source: BJPsych Open. 2022 Apr 19;8(3):e86. doi: 10.1192/bjo.2022.48 (PMC9059737; doi:10.1192/bjo.2022.48)
Supplement: Supplementary file 1 [file S2056472422000485sup001.docx]

**Table A.** Multivariable linear regression predicting symptoms of depression at completion of first year (square-root adjusted, sleep item removed) from locus of control and self-esteem at entry to first year, using data from Cohort 1.

|  | *∆R^2^* | *B* | *SE B* | *β* | *p* |
| --- | --- | --- | --- | --- | --- |
| Step 1 | 0.07 |  |  |  |  |
| Constant |  | 3.81 | 0.20 |  | < .001 |
| Female |  | 0.34 | 0.07 | 0.13* | < .001 |
| Lifetime mood disorder |  | 0.86 | 0.10 | 0.22* | < .001 |
| Step 2 | 0.22 |  |  |  |  |
| Constant |  | 4.82 | 0.26 |  | < .001 |
| Female |  | 0.12 | 0.06 | 0.05* | 0.04 |
| Lifetime mood disorder |  | 0.28 | 0.09 | 0.07* | < .01 |
| Internal LOC |  | 0.01 | 0.01 | 0.01 | .65 |
| External LOC |  | 0.02 | 0.01 | 0.10* | < .001 |
| Self Esteem |  | 0.06 | 0.01 | -0.28* | < .001 |
| Insomnia symptoms |  | 0.05 | 0.00 | 0.26* | < .001 |
| Adj. R^2^ for final model: 0.29 |  |  |  |  |  |

*B*: beta-value. *SE B*: standard error of beta-value. *β*: standardised beta-value. **p* < .05.

**Table B.** Multivariable linear regression predicting symptoms of depression at completion of first year (square-root adjusted, sleep item removed) from perfectionism and self-esteem at entry to university, using data from Cohort 2

|  | *∆R^2^* | *B* | *SE B* | *β* | *p* |
| --- | --- | --- | --- | --- | --- |
| Step 1 | 0.14 |  |  |  |  |
| Constant |  | 4.03 | 0.23 |  | < .001 |
| Female |  | 0.50 | 0.08 | 0.19* | < .001 |
| Lifetime mood disorder |  | 1.06 | 0.11 | 0.30* | < .001 |
| Step 2 | 0.28 |  |  |  |  |
| Constant |  | 3.97 | 0.36 |  | < .001 |
| Female |  | 0.18 | 0.07 | 0.07 | .01 |
| Lifetime mood disorder |  | 0.49 | 0.10 | 0.14 | < .001 |
| Personal standards |  | 0.02 | 0.01 | 0.05 | .10 |
| Evaluative concerns |  | 0.07 | 0.02 | 0.16 | < .001 |
| Self Esteem |  | -0.06 | 0.01 | -0.30 | < .001 |
| Insomnia symptoms |  | 0.04 | 0.00 | 0.22 | < .001 |
| Adj. R^2^ for final model: 0.41 |  |  |  |  |  |

*B*: beta-value. *SE B*: standard error of beta-value. *β*: standardised beta-value. **p* < .05.

**Table C.** Multivariable logistic regression predicting positive insomnia disorder screening at completion of first year from locus of control and self-esteem at entry to university, using data from Cohort 1

|  | B (SE) | OR | 95% CI |
| --- | --- | --- | --- |
| Step 1 |  |  |  |
| Constant | -1.57* (0.72) |  |  |
| Female | 0.50* (0.14) | 1.65 | 1.26 – 2.18 |
| Lifetime sleep disorder | 1.70* (0.34) | 5.45 | 2.87 – 10.93 |
| Step 2 |  |  |  |
| Constant | 2.86 (0.87) |  |  |
| Internal LOC | - 0.01 (0.03) | 0.99 | 0.94 – 1.05 |
| External LOC | 0.02 (0.01) | 1.02 | 0.99 – 1.05 |
| Self Esteem | - 0.10* (0.01) | 0.91 | 0.88 – 0.93 |

R^2^ = .07 (Hosmer-Lemeshow), .08 (Cox-Snell), .12 (Nagelkerke). Model χ^2^ (1) = 131.45,

p < .01. Note: **p* < .05.

**Table D.** Multivariable linear regression predicting positive insomnia disorder screen at completion of first year from perfectionism and self-esteem at entry to university, using data from Cohort 2.

|  | B (SE) | OR | 95% CI |
| --- | --- | --- | --- |
| Step 1 |  |  |  |
| Constant | 2.23* (0.99) |  |  |
| Female | 0.55* (0.18) | 1.74 | 1.23 - 2.49 |
| Lifetime sleep disorder | 1.99* (0.47) | 7.31 | 3.10 - 20.08 |
| Step 2 |  |  |  |
| Constant | 2.71 (1.30) |  |  |
| Personal Standards (CPQ) | -0.02 (0.03) | 0.98 | 0.93 - 1.03 |
| Evaluative Concerns (CPQ) | 0.14* (0.05) | 1.15 | 1.05 - 1.26 |
| Self Esteem | -0.09* (0.02) | 0.91 | 0.88 - 0.95 |

R^2^ = .12 (Hosmer-Lemeshow), .14 (Cox-Snell), .19 (Nagelkerke), Model χ^2^ (1) = 126.28,

*p* <.01. Note: **p* < .05.

**Table E.** Multivariable logistic regression predicting positive depression disorder screen at completion of first year from locus of control and self-esteem at entry to university, using data from Cohort 1

|  | B (SE) | OR | 95% CI |
| --- | --- | --- | --- |
| Step 1 |  |  |  |
| Constant | -1.39 (0.23) |  |  |
| Female | 0.40 (0.13) | 1.49 | 1.16 – 1.92 |
| Lifetime mood disorder | 1.42 (0.19) | 4.15* | 2.90 – 6.02 |
| Step 2 |  |  |  |
| Constant | -0.65 (0.63) |  |  |
| Internal LOC | 0.01 (0.03) | 1.01 | 0.96 – 1.07 |
| External LOC | 0.04 (0.01) | 1.04 | 1.01 – 1.07 |
| Self Esteem | -0.12 (0.02) | 0.88* | 0.86 – 0.91 |
| Insomnia symptoms | 0.09 (0.01) | 1.09* | 1.07 – 1.11 |

R^2^ = 0.18 (Hosmer-Lemeshow), 0.21 (Cox-Snell), 0.29 (Nagelkerke). Model χ^2^ (1) = 365.15. p < 01. Note: **p* < .05. **square-root adjusted.

**Table F.** Multivariable logistic regression predicting positive depression disorder screen at completion of first year from perfectionism and self-esteem at entry to university, using data from Cohort 2.

|  | B (SE) | OR | 95% CI |
| --- | --- | --- | --- |
| Step 1 |  |  |  |
| Constant | - 2.07 (0.34) |  |  |
| Female | 0.74* (0.18) | 2.11 | 1.48 – 3.05 |
| Lifetime mood disorder | 1.76* (0.24) | 5.83 | 3.68 – 9.53 |
| Step 2 |  |  |  |
| Constant | -3.07 (0.97) |  |  |
| Personal Standards | 0.04 (0.03) | 1.04 | 0.98 – 1.10 |
| Evaluative Concerns | 0.14* (0.05) | 1.15 | 1.04 – 1.27 |
| Self Esteem | -0.13* (0.02) | 0.88 | 0.84 – 0.92 |
| Insomnia symptoms | 0.09* (0.01) | 1.09 | 1.06 – 1.12 |

R^2^ = .27 (Hosmer-Lemeshow), .30 (Cox-Snell), .41 (Nagelkerke). Model χ^2^ (1) = 305.88,

p < .01. Note: **p* < .05. **square-root adjusted

**Table G.** Multivariable logistic regression predicting positive anxiety disorder screen at completion of first year from locus of control and self-esteem at entry to university, using data from Cohort 1

|  | B (SE) | OR | 95% CI |
| --- | --- | --- | --- |
| Step 1 |  |  |  |
| Constant | -1.65* (0.24) |  |  |
| Female | 0.57* (0.13) | 1.76 | 1.36 – 2.28 |
| Lifetime anxiety disorder | 1.15* (0.14) | 3.16 | 2.39 – 4.21 |
| Step 2 |  |  |  |
| Constant | -0.53 (0.56) |  |  |
| Internal LOC | 0.01 (0.03) | 1.00 | 0.95 – 1.06 |
| External LOC | 0.03* (0.01) | 1.03 | 1.00 – 1.06 |
| Self Esteem | -0.10* (0.01) | 0.91 | 0.88 – 0.93 |
| Insomnia symptoms | 0.08* (0.01) | 1.08 | 1.06 – 1.10 |

**square-root adjusted

**R^2^ = 0.16 (Hosmer-Lemeshow), 0.19 (Cox-Snell), 0.26 (Nagelkerke). Model χ^2^ (1) = 317.44, *p* < 01. Note: **p* < .05.**

**Table H.** Multivariable logistic regression predicting positive anxiety disorder screen at completion of first year from perfectionism and self-esteem at entry to university, using data from Cohort 2

|  | B (SE) | OR | 95% CI |
| --- | --- | --- | --- |
| Step 1 |  |  |  |
| Constant | -2.37* (0.35) |  |  |
| Female | 0.87* (0.19) | 2.39 | 1.65 – 3.51 |
| Lifetime anxiety disorder | 1.50* (0.18) | 4.46 | 3.13 – 6.41 |
| Step 2 |  |  |  |
| Constant | -4.32 (0.95) |  |  |
| Personal Standards (CPQ) | 0.10* (0.03) | 1.10 | 1.04 – 1.17 |
| Evaluative Concerns (CPQ) | 0.14* (0.05) | 1.16 | 1.05 – 1.27 |
| Self Esteem | -0.09* (0.01) | 0.92 | 0.88 – 0.96 |
| Insomnia symptoms | 0.09* (0.01) | 1.09 | 1.06 – 1.12 |

**R^2^ = 0.26 (Hosmer-Lemeshow), 0.29 (Cox-Snell), 0.39 (Nagelkerke). Model χ^2^ (1) = 291.50, *p* < 01. Note: **p* < .05.**
